# Inflammation‐Controlled Anti‐Inflammatory Hydrogels

**DOI:** 10.1002/advs.202206412

**Published:** 2022-12-29

**Authors:** Tina Helmecke, Dominik Hahn, Nadine Matzke, Lisa Ferdinand, Lars Franke, Sebastian Kühn, Gunter Fischer, Carsten Werner, Manfred F. Maitz

**Affiliations:** ^1^ Leibniz Institute of Polymer Research Dresden Institute of Biofunctional Polymer Materials Hohe Strasse 6 01069 Dresden Germany; ^2^ Max Planck Institute for Multidisciplinary Sciences 37077 Göttingen Germany; ^3^ Technische Universität Dresden Cluster of Excellence Physics of Life Center for Regenerative Therapies Dresden and Faculty of Chemistry and Food Chemistry Fetscherstraße 105 01307 Dresden Germany

**Keywords:** drug delivery, elastase, feedback control, hydrogel, inflammation

## Abstract

While autoregulative adaptation is a common feature of living tissues, only a few feedback‐controlled adaptive biomaterials are available so far. This paper herein reports a new polymer hydrogel platform designed to release anti‐inflammatory molecules in response to the inflammatory activation of human blood. In this system, anti‐inflammatory peptide drugs, targeting either the complement cascade, a complement receptor, or cyclophilin A, are conjugated to the hydrogel by a peptide sequence that is cleaved by elastase released from activated granulocytes. As a proof of concept, the adaptive drug delivery from the gel triggered by activated granulocytes and the effect of the released drug on the respective inflammatory pathways are demonstrated. Adjusting the gel functionalization degree is shown to allow for tuning the drug release profiles to effective doses within a micromolar range. Feedback‐controlled delivery of covalently conjugated drugs from a hydrogel matrix is concluded to provide valuable safety features suitable to equip medical devices with highly active anti‐inflammatory agents without suppressing the general immunosurveillance.

## Graphical abstract

A polymer hydrogel platform designed to release anti‐inflammatory molecules in response to the inflammatory activation of human blood is presented. Elastase from activated granulocytes releases covalently conjugated inhibitors against different steps of the inflammatory process from the hydrogel matrix. Suppression of the inflammation also terminates the elastase release from granulocytes and drug delivery ceases.

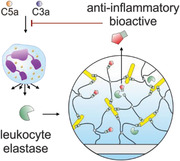

## 1 Introduction

Although current medical device design aims to minimize inflammation, the application of foreign materials in contact to human blood and tissues can still be associated with inflammatory reactions, resulting, for example, in the foreign body response to implants ^[^ ^1^ ^]^ or the neointimal thickening of restenosis in vascular stents.^[^ ^2^ ^]^ The activation of the complement system, a primary cause of biomaterial‐induced inflammatory reactions,^[^ ^3^ ^]^ activates granulocytes and monocytes via the complement fragments C3a and C5a. The activation of these leukocytes leads to degranulation and release of matrix‐degrading proteases, inflammatory cytokines, and reactive oxygen species, which support and drive the inflammatory process.^[^ ^4^ ^]^ Various nonsteroidal and steroidal anti‐inflammatory and antiproliferative drugs are applied to control inflammation and subsequent tissue proliferation.^[^ ^5^ ^]^ Other anti‐inflammatory molecules like cyclosporins, complement‐ and complement receptor inhibitors (compstatin Cp20 and PMX53)^[^ ^6^ ^]^, nuclear factor kappa B (NF*κ*B) pathway inhibitors,^[^ ^7^ ^]^ or the chemerin‐derived peptide C15^[^ ^8^ ^]^ are applied much more restrictively because the permanent activity of these highly potent agents can suppress important physiological reactions. Modulating the inflammatory response without suppression of mandatory immune functions is highly desired to support the tissue integration of implants.

Target‐site‐specific delivery of immunomodulatory molecules from implants can maximize the desired effect at reduced systemic drug exposure but still releases potentially harmful drugs independent of the actual need. Feedback‐controlled release systems providing drugs locally *and* in biologically fine‐tuned amounts can be expected to further reduce undesired side effects (in particular, overdosage and premature exhaustion of the delivery reservoir).^[^ ^9^ ^]^ Biomaterials providing such characteristics could be, for example, suitable for designing vascular stents that avoid restenosis, sensors and electrodes without isolating fibrous encapsulation, or dressings for chronic wounds suppressing excessive inflammatory response but maintaining the immune surveillance against pathogens.

Following a related concept, we recently introduced adaptively anticoagulant hydrogels, which release heparin at doses self‐adjusted to the actual coagulation status, representing a new paradigm for feedback‐controlled drug delivery systems to ensure the biocompatibility of blood‐contacting biomaterials.^[^ ^10^ ^]^ The system consists of biohybrid hydrogels made of four‐armed poly(ethylene glycol) (starPEG) and heparin, covalently crosslinked using peptides, which are selectively cleaved by activated coagulation factors. Cleavage of the linkers results in the release of heparin, which catalyzes the inhibition of the coagulation cascade and—in turn—ceases further hydrogel cleavage. These studies established peptide cleavage to trigger drug release from polymer hydrogels with high cargo capacity by specific proteases as a powerful platform to equip medical devices for adaptive biocompatibility. Within the current study, we have now expanded this approach to the adaptive control of immune reactions.

Leukocyte elastase (EC 3.4.21.37) is a serine protease, secreted by activated polymorphonuclear neutrophil granulocytes (PMN) and monocytes during inflammation. Leukocyte elastase cleaves peptides after small hydrophobic amino acids (V, C, and A) or the polar amino acid threonine (T).^[^ ^11^ ^]^ In inflamed tissue, it shows high activity in cleaving extracellular matrix components such as elastin, collagen types I–IV, fibronectin, and laminin. Its ability to process also multiple growth factors, cytokines, and receptors with activating or inactivating functions turns it into a regulator of inflammation.^[^ ^11^, ^12^ ^]^ Due to its multiple activities, elastase is under tight control by protease inhibitors, with a sub‐millisecond half‐life in plasma.^[^ ^13^ ^]^

Its elevated activity during inflammatory processes suggests elastase as a trigger for inflammation‐responsive release systems. Accordingly, Aimetti et al. recently reported neutrophil elastase‐cleavable hydrogels as effective release systems for sterically incorporated substances.^[^ ^14^ ^]^ Also, inhalable elastase‐responsive nanoparticles were developed for targeted drug delivery to inflammatory areas in the lung.^[^ ^15^ ^]^ All these systems are based on entrapping the bioactive molecules within the hydrogel network and release upon proteolytic cleavage of the hydrogel by elastase. Obviously, this concept requires sufficiently large drug molecules to avoid spontaneous release.^[^ ^14^, ^15^ ^]^ Alternatively, the small synthetic neutrophil exocytosis inhibitor Nexinhib20 was reported to be released in a nanoencapsulated form.^[^ ^15b^ ^]^ For this type of release system, downregulation of the cleaving enzyme cannot terminate the release, i.e., the release is not adaptive. Besides elastase, phosphatase, matrix metalloproteinase 2, or the oxidative environment of inflammatory tissue were suggested to trigger the release of anti‐inflammatory drugs, however, with lower inflammation‐selectivity.^[^ ^4^, ^9^, ^16^ ^]^

**Figure 1:**
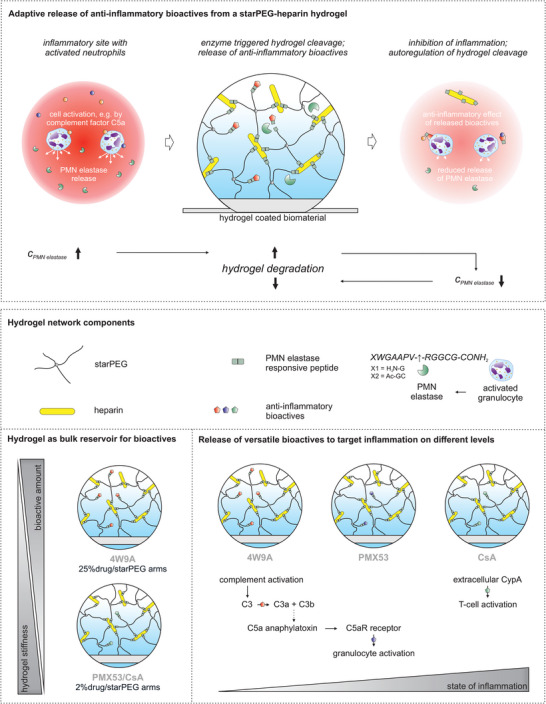
Schematic summary of the explored concept of inflammation‐responsive anti‐inflammatory hydrogels: Inflammatory processes activate granulocytes, followed by the release of cellular leukocyte elastase. A polymer hydrogel system of four‐armed poly(ethylene glycol) (starPEG) was crosslinked with heparin and contains linker peptides that are cleavable by leukocyte elastase in direct response to granulocyte activation. Anti‐inflammatory drugs are conjugated to the starPEG component of the hydrogel through the same cleavable linker peptide and released upon enzymatically controlled hydrogel cleavage. The amount of released drug can be adjusted via the degree of starPEG functionalization. Three different bioactives were applied to validate the adaptive anti‐inflammatory functionality of the hydrogel platform, targeting either the complement cascade, the leukocyte response to complement fragments, or the proinflammatory effects of extracellular cyclophilin A (CypA).

As an alternative to the sterically controlled release, we herein suggest the covalent conjugation of anti‐inflammatory compounds to a hydrogel matrix through enzymatically cleavable peptide linkers. As displayed in **Figure** **1**, the respective material consists of a starPEG–heparin network^[^ ^17^ ^]^ crosslinked and decorated with anti‐inflammatory drugs through an elastase‐cleavable linker‐peptide.^[^ ^10^, ^17^ ^]^ The versatility of the anti‐inflammatory hydrogel platform was elaborated for three inhibitors targeting different stages of the inflammatory process: a compstatin‐derived inhibitor of the complement pathway^[^ ^18^ ^]^ inhibits a humoral central inflammatory pathway, release of the complement C5a receptor inhibitor PMX53^[^ ^19^ ^]^ inhibits the cellular response to the complement cascade and an extracellular active cyclosporin A derivative inhibits the inflammatory response of other cells.

Elastase activity is the driver of tissue damage in many cases of inflammatory conditions in vivo^[^ ^12^, ^20^ ^]^ due to the concentrated and localized release from leukocytes. However, a realistic in vitro assessment of elastase‐responsive systems using whole blood is limited by the rapid inhibition of the protease in plasma, restricting previous in vitro studies to the analysis of release kinetics in buffered enzyme solutions.^[^ ^14^, ^15^ ^]^ In this study, this challenge is conquered by a two‐step incubation approach, where, in the first step, activated granulocytes induced drug release from the hydrogel in a plasma‐free environment to avoid protease‐inhibition by the excess of inhibitors in plasma. The release‐product of the hydrogel, containing the anti‐inflammatory drug, was applied in the second step to evaluate its effect on the respective inflammatory pathways.

## 2 Results and Discussion

### 2.1 Characterization of the Leukocyte Elastase‐Responsive Peptide and Conjugate

The elastase‐cleavable peptide, based on the recognition sequence AAPV^[^ ^14^ ^]^ (Table S1, Supporting Information), with the *C*‐ and *N*‐terminal extensions adapted for integration into the hydrogel, was conjugated at its *C*‐terminal cysteine group to four‐armed, maleimide‐terminated starPEG via Michael‐type addition. The peptide served as the linker both for hydrogel formation and drug conjugation. The conjugation slightly delayed the cleavage rate of the peptide from 0.36 to 0.15 s^−1^ (Figure S1, Supporting Information). This delayed cleavage may be attributed to the higher hydrodynamic radius of the starPEG–peptide conjugate and limited diffusion of the conjugated peptides compared to the free peptide.

The heparin component of the hydrogel matrix provides favorable additional anti‐inflammatory properties and interacts with the cleaving enzyme in different ways: Elastase has an anion binding site, which mediates binding to the DNA in neutrophil extracellular traps (NETs), to heparin and heparan sulfates, and also leads to accumulation at the hydrogel.^[^ ^21^ ^]^ Our previous study indicated that heparin affinity of the cleaving protease delays the cleavage of heparin–starPEG biohybrid hydrogels with protease‐cleavable linker peptides.^[^ ^10b^ ^]^ Additionally, direct inhibition of elastase activity by heparin binding has been reported.^[^ ^22^ ^]^ However, heparin also decreases the activity of *α*1‐antitrypsin, the main inhibitor of elastase.^[^ ^23^ ^]^

We probed the elastase activity at a wide range of heparin concentrations covering the released heparin concentrations almost up to the concentration within the hydrogel. Interference with the elastase activity with a U‐shaped suppression of elastase activity to about 50% was observed in the concentration range of heparin of 0.01–5 µg mL^−1^ (≈0.002–1 U mL^−1^) (Figure S2, Supporting Information).

### 2.2 Development of an Inflammation‐Responsive Drug Delivery Platform

Elastase‐cleavable starPEG–heparin hydrogels were formed using carbodiimide chemistry, as described before.^[^ ^10^, ^17^ ^]^ Carboxyl groups of heparin were linked to the N‐terms of the cleavable peptides at the starPEG arms, and the elastase responsiveness of these hydrogels was probed in buffered systems in a concentration range of the enzyme up to 150 nmol L^−1^, measuring the release of the matrix component heparin (Figure S3A, Supporting Information). This selected range of enzyme concentration covers the concentrations reported for wound exudates (15–100 nmol L^−1^).^[^ ^12^, ^20^ ^]^ The release correlated with the enzymatic activity; there was no spontaneous cleavage without elastase and no burst release. For elastase concentrations of 50 nmol L^−1^ and more, the release terminated after 4 h, whereas the cleavage with 15 nmol L^−1^ was linear up to 8 h. The effective cleavage of the hydrogels with the elastase responsive linkers confirms sufficient residual activity of elastase in the gel, despite inhibition by the high local heparin concentration and sterical hindrance of starPEG conjugated linkers.

The cleavage was further probed under more relevant conditions of activated granulocytes. Due to the high concentration of elastase inhibitors in plasma,^[^ ^13^ ^]^ isolated granulocytes were used. They were incubated with hydrogels with and without AAPV cleavable linker peptides for 2 h at 37 °C in the presence or absence of complement opsonized zymosan (OPZ) (1 mg mL^−1^) as an activator. No heparin release from hydrogels with starPEG directly conjugated to heparin, without cleavable peptides, was observed, excluding nonspecific hydrogel cleavage by hydrolysis or heparanase from granulocytes.^[^ ^24^ ^]^ The OPZ‐activated granulocytes induced almost two times the heparin release compared to resting granulocytes (Figure S3B, Supporting Information).

**Figure 2:**
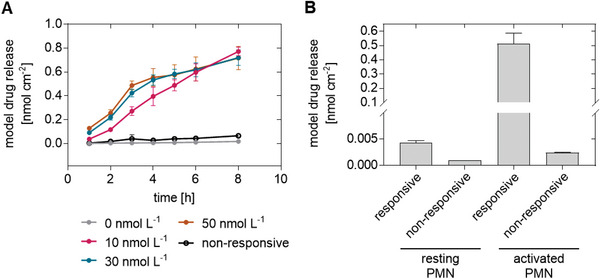
Release of a drug model compound (fluorescent dye Atto488) from a hydrogel. The model drug was conjugated to the amine term of the cleavable peptide at 2% of the starPEG arms. Nonresponsive control hydrogels were formed according to the same protocols using an amino‐terminated starPEG without the cleavable peptide. A) Molar release of the model compound by defined enzyme concentrations, rated to the hydrogel surface (mean ± SD of *n*  =  3). B) Release by resting and complement opsonized zymosan (OPZ)‐activated granulocytes normalized to the sample area (mean ± SD of *n* = 2).

The respective hydrogel was turned into a drug delivery system by conjugating anti‐inflammatory drugs to a defined fraction of starPEG arms with the AAPV peptide linker. Peptide cleavage releases the drug together with a peptide fragment. First, a fluorescent dye was conjugated to 2% of the starPEG arms as an easy‐to‐quantify model drug to validate the concept. Incubation of the hydrogel with the enzyme at different concentrations and with resting and OPZ‐activated granulocytes (**Figure** **2**) confirmed that there was no spontaneous drug release, and the release showed a gradual response with the enzyme concentration (Figure 2A). The activated cells released 0.51 ± 0.05 nmol cm^−2^ of the model drug from the hydrogel coating (Figure 2B). In the given test geometry, this release caused a concentration of about 2 µmol L^−1^ (1.6 ± 0.23 µmol L^−1^), which is in the therapeutic range of many anti‐inflammatory drugs. The hydrogel type was therefore found suitable to establish a responsive release platform for anti‐inflammatory drugs with the released drug concentration being adjustable by the fraction of drug‐conjugated starPEG arms.

Since the drug‐, respectively, dye‐functionalized starPEG arms are exempt from the hydrogel network structure, the impact of the drug conjugation on the mechanical gel properties was tested. Rheological analysis showed a minor decrease of stiffness *G*′ from 2.7 ± 0.96 to 2.5 ± 0.03 kPa for elastase‐responsive hydrogels after the model drug conjugation.

### 2.3 Responsive Anti‐Inflammatory Hydrogels

First, an inflammation‐responsive hydrogel release system for a compstatin‐based complement inhibitor was developed. The complement fragments C3a and C5a activate granulocytes, which release elastase, cleaving the hydrogel to deliver a complement inhibitor, forming a feedback control system.

The C3‐binding cyclic synthetic peptide compstatin was identified as an inhibitor of the complement cascade by Lambris et al.^[^ ^18b,c^ ^]^ More recent generations of the inhibitor are applied in clinical trials of acute and chronic inflammatory diseases.^[^ ^25^ ^]^ At systemic application, they proved efficient in preventing hemodialysis‐induced complement activation^[^ ^26^ ^]^ and reduced the inflammatory response to artificial implants.^[^ ^27^ ^]^

We synthesized the compstatin derivative 4W9A (Table S1, Supporting Information), which consists only of proteinogenic amino acids.^[^ ^18a^ ^]^ As the related analog Cp20 proved to tolerate *N*‐terminal extension with little loss of activity,^[^ ^18a^ ^]^ we synthesized 4W9A with an *N*‐terminal maleimide group to allow efficient conjugation by Michael‐type click chemistry to the cleavable peptide with an additional cysteine (C‐AAPV; see Table S1, Supporting Information).

**Figure 3:**
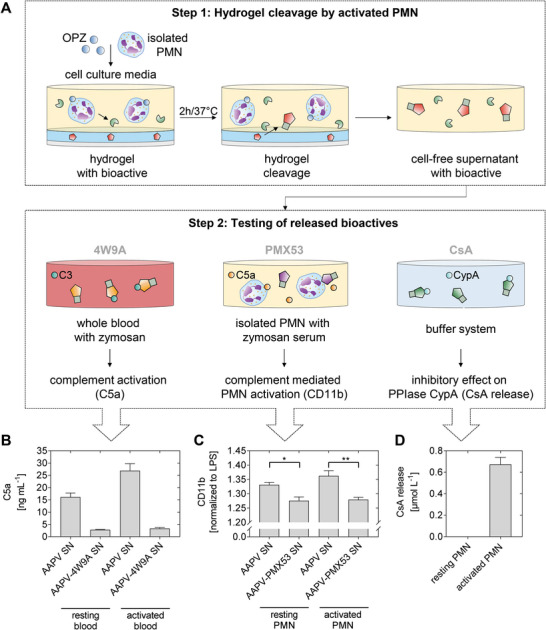
Cleavage of inflammation‐responsive hydrogels in an in vitro inflammation model and assessment of the anti‐inflammatory effects of the released drugs. A) For hydrogel cleavage, isolated polymorphonuclear granulocytes (PMN) are activated with complement‐opsonized zymosan (OPZ) in cell culture media and incubated with the hydrogel over 2 h at 37 °C. The cell‐free supernatant containing the released bioactive is used to test the anti‐inflammatory effect in the corresponding system. B) 4W9A: Measurement of complement activation in whole blood activated with zymosan. C) PMX53: Activation of isolated PMN by zymosan activated serum, measured as CD11b expression (*P < 0.05, **P < 0.01 in *t*‐test). D) CsA: Cyclosporine A (CsA) release measured by the inhibitory effect on CypA.

Probing its potency alone and in conjugation with the cleavage fragment of the peptide in a whole blood incubation assay with zymosan as complement activator (Figure S4, Supporting Information) indicated that the concentration of the inhibitor 4W9A should exceed the 2 µmol L^−1^ released from a responsive hydrogel with 2% of the starPEG‐arms drug‐functionalized (Figure 2A,B). Consequently, the rate of conjugated starPEG arms was elevated to ¼ (25%) for the 4W9A hydrogel without compensation of the lost crosslinks, resulting in a decrease of the hydrogel stiffness from 2.6 to 1.1 kPa. Upon incubation with OPZ‐activated granulocytes, the conjugated inhibitor had only a minor impact on hydrogel cleavage (Figure S5A, Supporting Information). Adding the supernatant containing the cleavage products of the hydrogel to whole blood followed by the complement activator zymosan (**Figure** **3**B) was shown to suppress the complement activation from 26.9 ± 2.9 to 3.3 ± 0.4 ng mL^−1^. Thus, the supernatant of nonstimulated granulocytes induced complement activation in blood, which was effectively suppressed by the complement inhibitor.

Complement reaction is initiated as a template‐dependent process that assembles individual complement factors on the activating surface. Consequently, a cascade inhibitor must be present directly at the activating surface to inhibit the complement system. The hydrogel coating shields the biomaterial surface and thereby passively restricts complement activation.^[^ ^10^ ^]^ In addition, the adaptive release of a complement inhibitor from the gel can locally target complement activation potentially resulting from other inflammatory response reactions.

Activated granulocytes can massively affect tissue remote from the activation site, as seen paradigmatically at hemodialysis therapy.^[^ ^28^ ^]^ The soluble complement fragment C5a is among the most potent activators and chemotactic stimuli of granulocytes via the G‐protein coupled C5a receptor (C5aR1 or CD88),^[^ ^29^ ^]^ its inhibitor PMX53 evolved as a promising anti‐inflammatory drug candidate and is already tested in clinical trials. The inhibitor is specific for the C5aR1 receptor and has no effect on leukocyte stimulation by cytokines, leukotriene B_4_, endotoxins, or formylated peptides.^[^ ^19^ ^]^ Therefore, a PMX53‐functionalized hydrogel was developed to prevent inflammatory damage of activated granulocytes locally and remotely from the site of the complement activation. No complement pathway inhibitor could afford this function.

PMX53 is derived from the C‐terminus of the C5a fragment. Therefore, *N*‐terminal sequence extension by a glycine spacer and a cysteine group appeared possible without loss of activity.^[^ ^19^, ^30^ ^]^ (Table S1, Supporting Information). The obtained peptide was linked to the elastase‐responsive hydrogel platform as an anti‐inflammatory feedback control system. The low IC_50_ concentration (IC_50_ = 20 nmol L^−1[^ ^19^ ^]^) allowed for a low functionalization degree at the hydrogel of only 2% of the starPEG‐AAPV‐arms. As for 4W9A, the inhibitor functionalization did not affect the cleavage rate of the hydrogel by activated granulocytes (Figure S5B, Supporting Information). In the two‐step incubation system, cells exposed to the supernatant of the PMX53‐containing hydrogels showed significantly less activation than cells conditioned with the supernatant of inhibitor‐free hydrogels. The activation of granulocytes for hydrogel cleavage in the first incubation step caused the release of proinflammatory mediators, such as TNF‐*α* or IL‐6, which were not further analyzed here. These mediators activated granulocytes in the second step and superposed all C5a‐dependent effects that were controlled by PMX53.

Cyclosporine A (CsA) has extracellular anti‐inflammatory activity by inhibition of the peptidyl‐*cis*–*trans*‐prolyl isomerase (PPIase) cyclophilin A (CypA), besides the intracellular immunosuppressive activity on lymphocytes via mTOR. CypA has proinflammatory effects on endothelial and smooth muscle cells and chemotactic effects on various leukocytes via CD147 activation.^[^ ^31^ ^]^ The CsA derivative MM284 inhibits extracellular CypA, providing anti‐inflammatory without immunosuppressive activity.^[^ ^32^ ^]^ For this study, the peptide drug was extended by the elastase‐cleavable sequence into MM623 (Table S1, Supporting Information) and conjugated to a maleimide‐terminated starPEG at the *C*‐terminal cysteine. The CsA–peptide conjugate MM623 inhibited CypA in a PPIase assay with characteristics comparable to MM284 (Table S2, Supporting Information). CsA inhibits calcineurin with an IC_50_ value of 0.149 µmol L^−1^. No inhibition was found for MM284 and MM623, up to 15 and 20 µmol L^−1^, respectively, corresponding to a 100‐fold increase. Thus, the PPIase and calcineurin assay suggest that MM623 is as anti‐inflammatory as MM284 and, likewise, does not elicit immunosuppressive effects.

Hydrogels were formed with 2% of the starPEG arms carrying the CsA derivative MM623. The incubation of the hydrogels containing MM623‐functionalized starPEG arms with resting and with zymosan‐activated granulocytes caused 10 times higher heparin release by the activated granulocytes, indicating hydrogel cleavage (Figure S5C, Supporting Information). Analysis of the CsA inhibitory activity in the supernatants by the PPIase assay confirmed that the drug was released only under inflammatory stimulation of granulocytes (Figure 3C).

The option of similarly conjugating different bioactive components without affecting the general setup of the hydrogel was considered a key advantage of our modular release system. Unlike known release systems relying on entrapment,^[^ ^14^, ^15^ ^]^ bioactives were covalently linked to the hydrogel matrix via the elastase‐responsive peptide at the starPEG arms, allowing for the precise bio‐adaptive release. However, the selected drug molecules need to contain reactive sites suitable for selective conjugation, as a fragment of the cleavable peptide remains with the released product and effects on the activity as well as the solubility and membrane permeability of the drug have to be considered. Peptide‐type drugs frequently fulfill these requirements or can be modified with cysteine and maleimide groups to enable their conjugation by Michael‐type addition.

Besides the anticoagulant properties, the structural component heparin in the hydrogel has desired anti‐inflammatory activity by binding the inhibitory factor H of the complement cascade and sequestration of inflammatory mediators.^[^ ^33^ ^]^ In our investigated system, the release of anti‐inflammatory peptide drugs is always accompanied by additional anticoagulant and anti‐inflammatory properties resulting from the release of heparin. In applications where the anticoagulant activity of heparin would be undesired, desulfated heparin as a polyanion without anticoagulant activity or carboxyl‐terminated starPEG molecules can be used in the hydrogel design instead of heparin.^[^ ^34^ ^]^

We showcased the efficacy of the adaptive, elastase‐responsive hydrogel‐based release system with three different peptide‐type drugs, addressing different scenarios. The complement inhibitor 4W9A targets an initial stage of inflammation and has mainly local activity; therefore, the coating of vascular stents by the respective hydrogel release system would be an attractive application. The complement receptor inhibitor PMX53, however, is active at a later stage of inflammation and allows for targeting mobile blood cells to prevent remote tissue damage. Finally, the cyclosporin‐derivative MM623 targets cyclophilin‐dependent local inflammatory processes and suppresses leukocyte recruitment in inflammatory processes.^[^ ^35^ ^]^

Importantly, the introduced adaptive anti‐inflammatory hydrogel release system can be expanded to apply a broad range of other anti‐inflammatory molecules, and combinations thereof. For instance, the incorporation of alternative complement inhibitors with different profiles,^[^ ^30a^ ^]^ chemerin‐derived peptides resolving inflammation in a receptor‐mediated way^[^ ^8b^ ^]^ and membrane translocation peptide sequences, guiding, e.g., inhibitor peptides for the intracellular NF*κ*B pathway (I*κ*B kinase inhibitors)^[^ ^36^ ^]^ seem worthwhile investigating. Beyond that, using the modularity of the hydrogel platform to create multimodal combinations of complementary adaptive release systems, interactively controlling different inflammation mechanisms as well as coagulation activation,^[^ ^10^ ^]^ is considered a highly attractive perspective.

## 3 Conclusion

While living matter often displays dynamic adaptation of functional characteristics, only a few man‐made materials are feedback‐controlled adaptive systems. Herein, we report an elastase‐sensitive hydrogel platform for the feedback‐controlled delivery of immunomodulatory drugs that is suitable to equip medical devices with anti‐inflammatory properties without the risk of losing the general immunosurveillance due to drug overdosage.

Three demonstrator hydrogels, releasing different anti‐inflammatory drugs, were synthesized, and functionally characterized to show the versatility of the system. The drugs inhibit either the complement cascade, a complement receptor, or cyclophilins, highlighting the possibility of targeting different stages of the inflammation process. Two of the release systems, 4W9A and PMX53, address the complement system. The compstatin derivative 4W9A inhibits the propagation of the complement cascade at the site of the origin, whereas PMX53 suppresses the response of leukocytes to complement activation. The cyclosporin MM623 has complement‐independent anti‐inflammatory effects on native tissue cells.

Elastase inhibitors impede hydrogel cleavage in activated whole blood and generally restrict in vitro tests to cleavage studies in buffer solutions. Therefore, an advanced assay was developed where elastase, secreted from leukocytes after activation by complement or other stimuli, acts as the trigger to release the inhibitors. With this assay, we were able to demonstrate that both complement‐targeting systems can establish a feedback‐control system, in which the release product specifically inhibits the stimulus of the activation.

The results obtained in this study, in particular, the validation data obtained by a novel, advanced in vitro model, suggest the introduced adaptively anti‐inflammatory hydrogel platform system as highly promising for the functionalization of medical implants. In a next step, the suitability of the materials will be therefore assessed in animal models. Beyond that, the complementarity of the different elastase‐triggered anti‐inflammatory functions, as well as the heparin release controlled by coagulation enzyme‐cleavable peptide linkers, will be used in combinatorial screening approaches to identify multifunctional, feedback‐regulated materials providing unprecedented biocompatibility.

## 4 Experimental Section

The detailed methods are provided in the supporting information.

### Synthesis of Responsive Peptides and Anti‐Inflammatory Bioactives

Different variants of the leukocyte elastase responsive peptide, as well as the cyclic peptide inhibitor 4W9A (Table S1, Supporting Information), were synthesized by standard Fmoc solid‐phase peptide synthesis, using a rink amide resin (Iris Biotech GmbH, Marktredwitz, Germany) as solid phase on a fully automated microwave peptide synthesizer (CEM Corporation, Matthews, USA). Postsynthesis peptide modifications (e.g., acetylation, maleimide functionalization, and cyclization) were directly performed on the resin as described in the supporting information.

The extracellularly acting cyclosporine A (CsA) MM623 (CsA‐(dE)_6_‐GKGAAPV‐↑‐GGC‐CONH_2_) was synthesized as a CsA–peptide conjugate for which MM284 was used as CsA analog. The synthesis of MM284 has already been described by Malešević.^[^ ^32^ ^]^ The details of the synthesis are described in the supporting information.

The cyclic peptide PMX53 derivative Ac‐CGF[Orn‐P‐Cha‐WR] was custom synthesized by Peptide 2.0, Chantilly, USA, with a documented mass identity of 1056.44 g mol^−1^ and a purity of 96.57% (HPLC).

### starPEG–Peptide Conjugates with Fluorescent Model Drug and Anti‐Inflammatory Bioactives

starPEG–peptide–drug conjugates with 2% of the starPEG–peptide arms functionalized with the drug were prepared for the fluorescent drug model, PMX53 derivative, and CsA. 25% of starPEG–peptide arms were coupled with 4W9A. For the synthesis of these conjugates, both the maleimide‐terminated four‐armed starPEG and the leukocyte elastase responsive peptide were separately dissolved in acetonitrile/H_2_O (50/50% v/v), mixed, and diluted to 8%–10% final solid content in phosphate‐buffered saline (PBS). Thereby the responsive peptide was used in 10% excess to the targeted amount of coupled starPEG arms. Peptide conjugation by Michael‐type addition was carried out at pH 7.5–8 for several hours under argon stream with subsequent purification by reversed‐phase HPLC (Agilent, Santa Clara, USA).

The strategies for the conjugation of the fluorescent model drug or immunomodulatory bioactives had to be adapted for different functional groups of the molecules, as outlined for each molecule and described in detail in the supporting information.

### Formation and Mechanical Characterization of Stable and Responsive Hydrogels

Stable and cleavable hydrogels were prepared by carbodiimide chemistry, as described previously.^[^ ^10a^ ^]^ Briefly: Carboxyl groups of heparin were activated by *N*‐(3‐dimethylaminopropyl)‐*N*′‐ethyl‐carbodiimid/*N*‐hydroxysulfosuccinimid (EDC/s‐NHS) for 15 min on ice. Subsequently, an amino‐terminated four‐arm starPEG or its conjugate with cleavable peptides and the drug was dissolved and added to the activated heparin. The gel solution was pipetted onto poly(ethylene‐*alt*‐maleic acid anhydride) copolymer (PEMA) precoated glass slides,^[^ ^37^ ^]^ immediately covered with a Sigmacote hydrophobized coverslip and polymerized overnight in a humidified atmosphere at room temperature. Finally, hydrogels were progressively washed and swollen for 24 h in PBS to remove residual EDC/s‐NHS or unbound reaction educts. Swollen hydrogels contain by mass about 2.5% starPEG, 3.5% heparin, 1% of the cleavable peptide, and the rest water, with possible variation due to different crosslinking degrees.

For mechanical characterization, hydrogels of 67 µL gel solution between two 9 mm diameter Sigmacote hydrophobized coverslips were prepared, resulting in 1 mm height after PBS swelling. The storage modulus *G*′ was determined by oscillating measurements on a rotational rheometer, as described before.^[^ ^10a^ ^]^ Comparable mechanical properties of the different gels were ensured by different heparin to starPEG ratios for stable (1:1.5/*ɣ* = 1.5) and responsive gels (1:1/*ɣ* = 1).

### Hydrogel Cleavage in an In Vitro Inflammation System

For hydrogel cleavage by granulocyte‐derived elastase, a serum‐free in vitro inflammation system using activated isolated granulocytes was developed. Hydrogels were prepared by polymerizing 21 µL hydrogel–precursor solution on 25 mm diameter PEMA coated glass slides and swelling in PBS for 24 h. Human whole blood was freshly drawn from two voluntary ABO compatible donors, anticoagulated with 5 IU mL^−1^ heparin after informed consent. Blood cell count and a test for C‐reactive protein (Diagnostica Nord, Schwerin, Deutschland) were performed to exclude acute inflammation in the donors. The study was approved by the ethic commission of the Sächsische Landesärztekammer (EK‐BR‐24/18‐1).

Granulocytes were isolated from pooled blood by sequential density gradient centrifugation,^[^ ^38^ ^]^ using a double density gradient of Polymorphprep and Lymphoprep, followed by a Percoll gradient centrifugation step. Isolated granulocytes were suspended in RPMI medium substituted with 2% BSA.

For artificial cell activation, zymosan A was complement opsonized by swelling in PBS at 95 °C for 1.5 h, followed by incubation in recalcified citrate plasma for 30 min at 37 °C and two subsequent washing steps in PBS. The hydrogel‐coated glass slides were used as top and bottom of incubation chambers, where 2 mL blood contact 6.3 cm^2^ hydrogel surface.^[^ ^39^ ^]^ Blood or isolated cells with 1 mg mL^−1^ complement opsonized zymosan (OPZ) were incubated for 2 h at 37 °C under constant overhead rotation. The cell activation was confirmed by elevated CD11b expression at the OPZ activated cells compared to nonactivated cells. Hydrogel cleavage was followed by heparin measurement in the supernatant using the Factor‐Xa‐based chromogenic assay Chromogenix Coamatic Heparin (Chromogenix, Milano, Italy).

### Analysis of hydrogel released drug concentration and inhibitory effect

The amount of Atto488 released from the hydrogel as a model drug was determined by fluorescence measurement at *λ*_ex._ = 485 nm/*λ*_em._ = 535 nm against an Atto488 standard. The inhibitory effect of soluble as well as hydrogel‐released compstatin 4W9A was tested in whole blood, where the complement system was activated with 10 µg mL^−1^ nonopsonized zymosan for 30 min at 37 °C. The supernatant of cleaved PMX53 conjugated hydrogels was exposed to isolated granulocytes in the presence of 5% zymosan‐activated serum for 20 min at 37 °C. Cell activation was determined by flow cytometry using CD11b‐VioBlue staining (Biolegend) for isolated granulocytes and additionally CD14‐APC and CD41a‐FITC (both BD Biosciences, New Jersey, USA) for whole blood samples. Leukocyte elastase and C5a concentrations were determined using the commercial ELISA kits Hycult human elastase ELISA (Hycult Biotech, Uden, Netherlands) and C5a micro ELISA (DRG, Marburg, Germany) according to the manufactures instructions.

### PPIase Inhibition

The peptidyl‐prolyl *cis*–*trans* isomerase (PPIase) activity of cyclophilin A (CypA) in the presence of different concentrations of CsA derivatives (MM284 and MM623) was determined as published before.^[^ ^40^ ^]^ From the activity against the inhibitor concentrations, the IC_50_ and the *K*_i_ value were determined with nonlinear curve fitting according to the models of Morrison^[^ ^41^ ^]^ and Murphy.^[^ ^42^ ^]^ Subsequently, supernatants from the release system were added to the PPIase assay and the concentration of CsA derivatives were determined.

### Calcineurin Activity Assay (CaN)

Calcineurin activity assay was described by Nacev.^[^ ^43^ ^]^ CsA derivative/CypA (15 µmol L^−1^), 1 U µL^−1^ CaN, and 300 nmol L^−1^ calmodulin were preincubated at 30 °C for 15 min in a 96‐well plate. The dephosphorylation reaction was started by addition of RII phosphopeptide, malachite green reagent was added after 30 min at 30 °C, and the absorbance was measured at 610 nm (Versamax plate reader).

### Statistical Assessment

All experiments were performed at least in triplicate, unless indicated differently. Data are presented as mean ± standard deviation (mean ± SD). The applied statistical tests are indicated in the figure captions.

## Conflict of Interest

The authors declare no conflict of interest.

## Supporting information

Supporting Information

## Data Availability

The data that support the findings of this study are openly available in Zenodo at https://doi.org/10.5281/zenodo.7296177, reference number 7296177.
