# Supplementary material for: Inflammation‐Controlled Anti‐Inflammatory Hydrogels
Source: Adv Sci (Weinh). 2022 Dec 29;10(7):2206412. doi: 10.1002/advs.202206412 (PMC9982591; doi:10.1002/advs.202206412)
Supplement: Supplementary file 1 — Supporting Information [file ADVS-10-2206412-s001.pdf]

Supporting Information

Inflammation-controlled anti-inflammatory hydrogels

Tina Helmecke, Dominik Hahn, Nadine Matzke, Lisa Ferdinand, Lars Franke, Sebastian Kühn, Gunter Fischer, Carsten Werner, Manfred F. Maitz

Supporting Figures

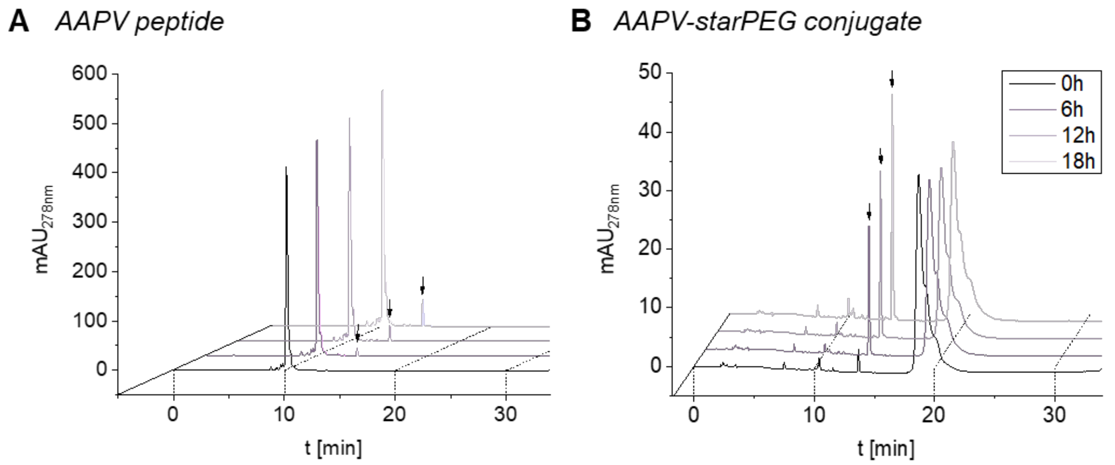

Figure S1: Cleavage of the peptide H_2N-GWGAAPV↑RGGCG-$CONH_2$ and its conjugate to four armed starPEG by leukocyte elastase. The elastase-sensitive AAPV peptide at a concentration of 7 mmol L^{-1} or 0.7 mmol L^{-1} starPEG-AAPV peptide conjugate were incubated with 20 nmol L^{-1} leukocyte elastase over 18 h at 37°C. The educt cleavage was followed by reversed-phase HPLC according to an established protocol.[10a] Chromatograms after 0, 6, 12, 18 h cleavage are presented in cascade form. (A) Formation of a single cleavage product (arrows) by AAPV peptide cleavage with higher hydrophobicity than the educt. (B) Cleavage of starPEG-AAPV conjugate, resulting in the formation of a cleavage product (arrows) with higher hydrophilicity compared to the educt. AU: Absorbance Units at 278 nm.

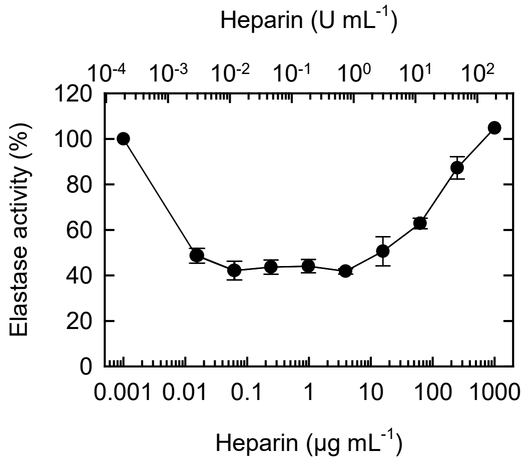

Figure S2: Inhibition of leukocyte elastase by heparin. A concentration of 10 nmol L^{-1} human leukocyte elastase was used to cleave 250 µmol L^{-1} chromogenic substrate in the presence of different heparin concentrations. The substrate cleavage was monitored photometrically at a wavelength of 405 nm by the release of *p*-nitroaniline from the chromogenic substrate methoxysuccinyl-Ala-Ala-Pro-Val-*p*NA over 30 min. Values were normalized to a control sample without heparin; mean ± SD of n=2.

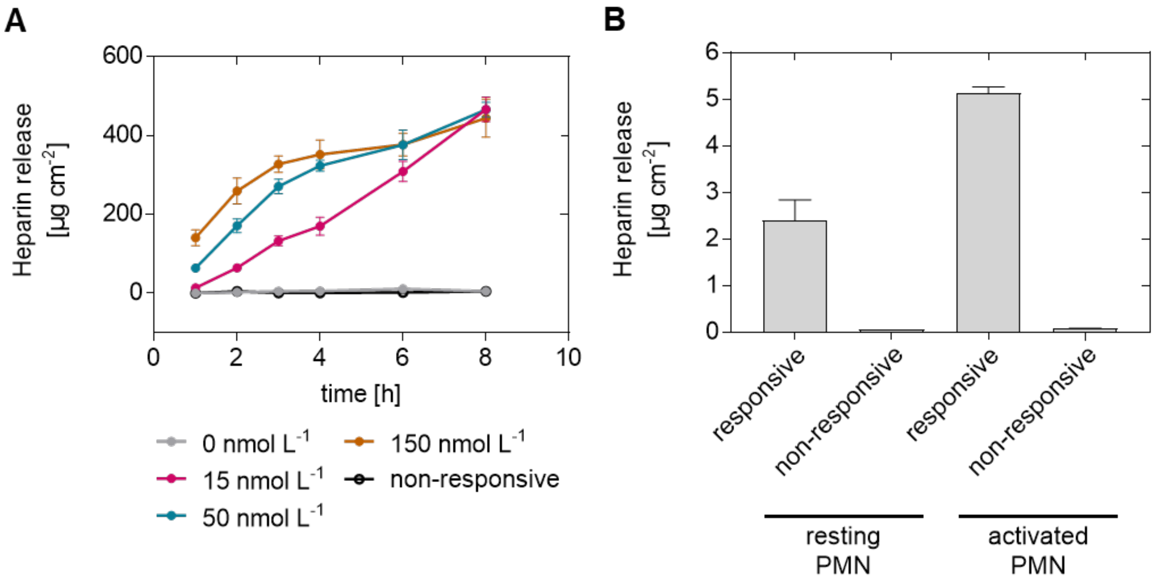

Figure S3: Cleavage of starPEG-heparin hydrogels determined by the release of the matrix component heparin. (A) Cleavage by the isolated enzyme (mean ± SD of n = 3) (B) Cleavage by stimulated granulocytes (mean ± SD of n = 2)

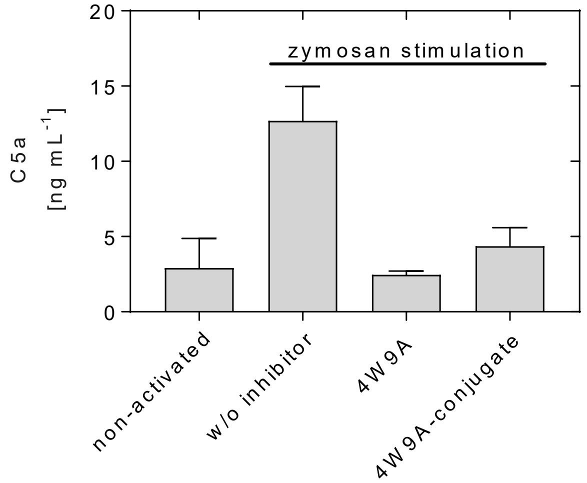

Figure S4: Evaluation of the complement inhibitory effect of the pure inhibitor 4W9A and its conjugate to the AAPV cleavage fragment (amidated form: Ac-GCWGAAPV-$CONH_2$) at a concentration of 10 µmol L^{-1}. The complement system was activated with 10 µg mL^{-1} zymosan. C5a concentrations presented as mean ± SD of n = 3.

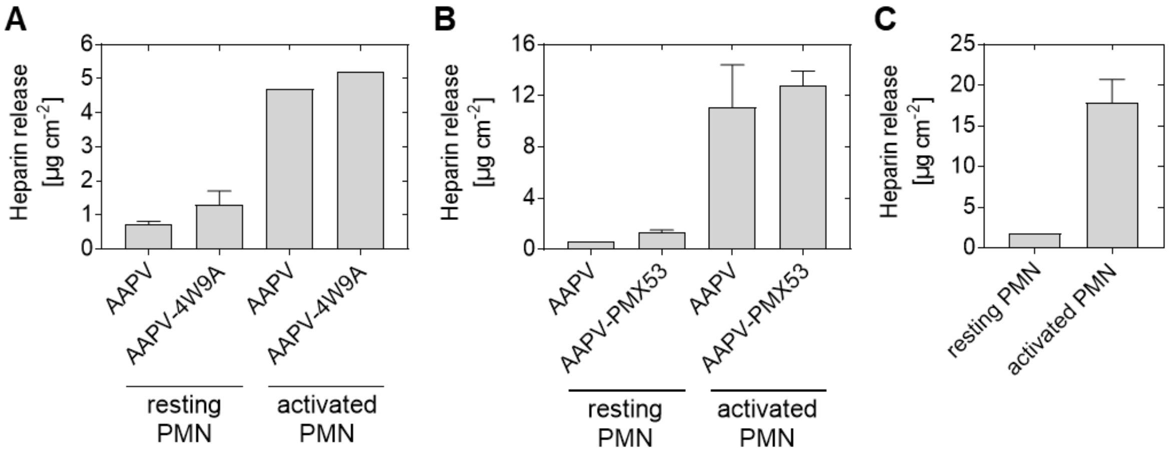

Figure S5: Evaluation of the inflammation-triggered complement-inhibitory hydrogel. In a first step, elastase-responsive hydrogels (AAPV) with and without inhibitor were incubated with resting or OPZ-stimulated granulocytes. (A) Heparin release complement inhibitory hydrogels (4W9A) as a marker of hydrogel cleavage. (B) Heparin release of hydrogels with complement receptor inhibitor PMX53 (C) Heparin release from hydrogels containing the cyclosporine MM623 (mean ± SD of n = 2).

Table S1: Peptides in this study. ↑ indicates the cleavage site by elastase-mediated cleavage. The amino acid O in PMX53 indicates L-ornithine, dCha indicates cyclohexyl-D-alanine. 4W9A was cyclized by a disulfide bond, and PMX53 was backbone-to-side chain cyclized.

Label	Description	Sequence
AAPV	Elastase-cleavable linker peptide	H_2N-GWGAAPV-↑-RGGCG-$CONH_2$
C-AAPV	Elastase-cleavable linker peptide with an additional N-terminal cysteine	Ac-GCWGAAPV-↑-RGGCG-$CONH_2$
4W9A	Complement pathway inhibitor	Mal-I[CVWQDWGAHRC]-T-$CONH_2$
PMX53	C5a receptor blocker	Ac-CGF-[OPdChaWR]
MM623	CsA derivative with elastase-cleavable sequence	CsA-(DGlu)$_6$-GKGAAPV-↑-GGCG-$CONH_2$

Table S2: Overview of the inhibition of PPIase activity and CaN activity by CsA, MM284 and MM623

Compound	PPIase		CaN
	IC_{50} (nmol L^{-1})	K_i (nmol L^{-1})	IC_{50} (µmol L^{-1})
CsA	3.09 ± 0.77	5.22 ± 1.04	0.149 ± 0.034
MM284	9.10 ± 1.43	6.42 ± 0.88	>15
MM623	4.63 ± 1.00	1.55 ± 0.59	>20

Supporting method section

S1.1. Chemicals

The amino acids used for peptide synthesis Fmoc-Ala*H_2O, Fmoc-Arg(Pbf)-OH, Fmoc-Asp (tBu)-OH, Fmoc-Cys(Acm)-OH, Fmoc-Cys(StBu)-OH, Fmoc-Cys (Trt)-OH, Fmoc-Gln(Trt)-OH, Fmoc-Gly-OH, Fmoc-His(Trt)-OH, Fmoc-Ile-OH, Fmoc-Pro-OH*H_2O, Fmoc-Thr(tBu)-OH, Fmoc-Trp(Boc)-OH, Fmoc-Val-OH as well as N,N-dimethylformamide (DMF), trifluoroacetic acid (TFA), diethyl ether (DEE), ethyl cyano(hydroxyimino)acetate (Oxyma), piperidine, N,N-diisopropylethylamine (DIPEA), 1,4-dithiothreitol (DTT), N,N,N′,N′-tetramethyl-O-(1H-benzotriazol-1-yl)uronium hexafluorophosphate (HBTU) and 1-ethyl-3-(3-dimethylaminopropyl) carbodiimide (EDC) were purchased from Iris Biotech, Marktredwitz, Germany. N,N′-diisopropylcarbodiimide (DIC), tris(2-carboxyethyl)phosphine (TCEP) and phenol were from Carl Roth, Karlsruhe, Germany; dichloromethane (DCM) and acetonitril (AcN) from Acros Organics, Geel, Belgium; triisopropylsilane (TIS) and N-hydroxysuccinimide ester were from Alfa Aesar, Haverhill, USA. N-hydroxysulfosuccinimide sodium salt (s-NHS), 2-amino-2-(hydroxymethyl)-1,3-propanediol (Tris), sodium chloride (NaCl), bovine serum albumin (BSA), 1-hydroxybenzotriazole (HOBt), phosphate-buffered saline tablets (PBS), Sigmacote®, Atto488-NHS fluorescence dye and Zymosan A were purchased from Sigma-Aldrich, Munich, Germany. Thallium(III)trifluoroacetate (TTFA) was from Tokyo Chemical Industry, Tokyo, Japan and maleimide- and amino-terminated four-armed starPEG from JenKem Technology, Plano, USA. Human neutrophil elastase, the chromogenic substrate MeOSuc-Ala-Ala-Pro-Val-pNA and heparin sodium salt for hydrogel preparation were purchased from Merck/Calbiochem, Darmstadt, Germany; heparin sodium for blood experiments was from ratiopharm, Ulm, Germany; Polymorphprep and Lymphoprep from Axis shield, Oslo, Norway; Percoll from

GE Healthcare Biosciences AB, Uppsala, Sweden and RPMI media from PAN Biotech, Aidenbach, Germany. FACS antibody CD11b-VioBlue was purchased from Biolegend, San Diego, USA, CD14-APC and CD41a-FITC were obtained from BD Biosciences, New Jersey, USA.

S1.2. Synthesis of responsive peptides and anti-inflammatory bioactives

Synthesis of the leukocyte elastase responsive peptides AAPV and C-AAPV, as well as the cyclic peptide inhibitor 4W9A (Table S1) was performed by microwave-assisted Fmoc solid phase peptide synthesis. Amino acids were coupled using DIC and Oxyma in DMF as activators at 55°C and subsequently deprotected in piperidine solution (20% v/v in DMF) at 70 °C. Peptide cleavage from the resin as well as removal of side chain protection groups was performed in TFA/phenol/TIS/water (87.5/5/2.5/5% v/v) for 4h. To prevent the formation of disulfide bonds in 4W9A, 2.5% of TFA were substituted by DTT. Peptides were precipitated in DEE, filtered, and purified by preparative RP-HPLC (Agilent, Santa Clara, USA).

The identity and purity of the synthesized peptides were determined by HPLC-MS on a 1260 infinity LC System coupled with an Agilent 6230 time-of-flight LC/MS instrument (Agilent technologies, Santa Barbara, USA) as described elesewhere.[44] The molecular weight of the AAPV and C-AAPV peptides was confirmed as 1085.53 g mol^{-1} and 1318.60 g mol^{-1} with purities of 99% and 85.8%, respectively. The yield of 4W9A was 8.5%, the molecular weight was confirmed by mass spectroscopy as 1613.71 g mol^{-1} with a purity of 84.8%.

For certain products, peptide modifications were performed directly after peptide synthesis on the rink-amide resin before cleavage.

The cleavage of the pure peptide and the conjugate were analyzed by reversed-phase HPLC (**Figure S1**).

Chromatographic sample separation demonstrated the formation of a single product peak upon enzymatic hydrolysis. AAPV peptide cleavage resulted in the formation of a cleavage product with higher hydrophobicity than the educt (retention $time_{peptide}$ = 9.9 min, retention $time_{product}$ = 13.4 min). In contrast, the cleavage product of the starPEG-AAPV conjugate appeared more hydrophilic compared to the educt (retention $time_{conjugate}$= 18.6 min, retention $time_{product}$= 13.5 min). An expected second product peak was not detectable, revealing similar physico-chemical properties of this compound to the educt or the first product (**Figure S1**). After 18 h, 0.35 ± 0.03 mmol L^{-1} (5.0% ± 0.5%) of the free peptide and 0.082 ± 0.017 mmol L^{-1} (11.8% ± 2.4%) of the conjugate were hydrolyzed, resulting in a substrate conversion of 5.28 ± 0.51 nmol L^{-1} s^{-1} for the free peptide and 1.25 ± 0.26 nmol L^{-1} s^{-1} for the conjugate. In the first 180 min, the maximum substrate cleavage of 7.23 nmol L^{-1} s^{-1} was determined, resulting in a cleavage rate of 0.36 s^{-1}. The starPEG conjugation hindered the cleavage by the peptide, and the cleavage rate decreased to 0.15 s^{-1}.

Cyclization of compstatin 4W9A

To enable a cyclization of 4W9A, the cysteine amino acids used for peptide synthesis were protected by acetamidomethyl (Acm). To cleave the protection groups the peptide resin was preswollen in DMF for 40 min. Subsequently, a 2 fold excess of TTFA was added. After 3h the reaction mixture was removed by filtration and the peptide resin washed in DMF.

N-terminal acetylation of the leukocyte elastase responsive peptide C-AAPV and compstatin 4W9A

To prevent side reactions of the N-terminus upon further conjugation of the cleavable peptide C-AAPV, the free amino group was acetylated by a stepwise incubation of the resin with acetic anhydride for 10 min and 15 min, followed by DMF and DCM wash. The same acetylation step was applied also to 4W9A for evaluation of the inhibitor performance.

N-terminal maleimide functionalization of compstatin 4W9A

Since the cyclic peptide inhibitor compstatin 4W9A should be bound to the leukocyte elastase responsive peptide (C-AAPV) by Michael-type addition, a reactive maleimide moiety was introduced to the 4W9A N-terminus. For this purpose, the peptide resin was swollen in DMF for 30 min. A 4-fold excess of maleimido propionic acid to the peptide was dissolved in DMF and activated by HOBt/HBtU (3.5 fold excess to acid). After addition of DIPEA (7 fold excess to acid) the reaction mixture was added to the pre-swollen peptide resin. After 30 min the reaction mixture was removed by filtration, and the peptide resin washed in DMF.

Synthesis of the peptide conjugate MM623

The extracellularly acting cyclosporine A (CsA) MM623 (CsA-(DE)$_6$-GKGAAPV-↑-GGC-$CONH_2$) was synthesized as a CsA-peptide conjugate for which MM284 was used as CsA analog. The synthesis of MM284 has already been described by Malešević.[32] The peptide was synthesized sequentially using standard Fmoc chemistry on rink-amide resin. For the

amino acid Cys Fmoc-Cys(StBU) was used. In the final step of peptide extension, MM284 was introduced via HATU/HOBt in DMF. The construct was cleaved from the resin with 95 % TFA for 30 min, TFA was removed in vacuum, the peptide was purified by preparative HPLC and the cysteine moiety was deprotected reductively with 4 equiv. TCEP in MeOH/ACN/water. Finally, the product was again purified by preparative HPLC and lyophilized. Next to MM623, MM622 (CsA-(DE)$_6$-GKGAAPV-OH) was synthesised as a control in the same manner using a chlortrityl-resin.

The purity of the final products was determined to >95% using a Dionex analytical HPLC unit in combination with a photodiode arraydetector and a Vydac reverse phase analytical column (C18, 5 μm, 4.6 mm × 250 mm) in conjunction with a Surveyor MSQ (Thermo Finnigan)mass spectrometer. MM623 - ESI (m/z): 2894.3 $[M + H]^+$ and MM622 - ESI (m/z): 2619.4 $[M + H]^+$.

S1.2. Supplement – fluorescent drug model and bioactive conjugation

starPEG-peptide-drug conjugates with 2% of the starPEG-peptide arms functionalized with the drug were prepared for the fluorescent drug model, PMX53 derivative, and CsA. 25% of starPEG-peptide arms were coupled with 4W9A. For the synthesis of these conjugates, both the maleimide-terminated four-armed starPEG and the leukocyte elastase responsive peptide were separately dissolved in acetonitrile/H_2O (50/50% v/v), mixed, and diluted to 8-10% final solid content in PBS. Thereby the responsive peptide was used in 10% excess to the targeted amount of coupled starPEG arms. Peptide conjugation by Michael-type addition was carried out at pH 7.5-8 for several hours under argon stream with subsequent purification by reversed-phase HPLC (Agilent, Santa Clara, USA).

Coupling of fluorescent drug model

A starPEG-peptide conjugate with full starPEG arm saturation by the leukocyte elastase responsive AAPV peptide was synthesized and purified as described in the Methods section. For the conjugation of the fluorescent drug model to the terminal NH_2 of the responsive peptide, 15 mmol L^{-1} starPEG-peptide conjugate were reacted with 1.2 mmol L^{-1} Atto488-NHS in 0.1 mol L^{-1} sodium bicarbonate buffer (pH 8.3) for 1 h, targeting a final labeling degree of 2% starPEG-peptide arms. Subsequently, the reaction mixture was directly used for hydrogel formation. As control for stable hydrogel formation, four-armed starPEG amine was labeled with the same principle.

Coupling of compstatin 4W9A

To enable the binding of maleimide functionalized compstatin 4W9A, a leukocyte elastase responsive peptide with an additional, StBu protected cysteine at the N-terminal site of the peptide cleavage sequence was used for starPEG-peptide conjugate formation. In a first step, four-armed maleimide-terminated starPEG was reacted with the equal molar ratio of the AAPV-Cys peptide for 1 h, to couple 25% of starPEG arms to the peptide. The pH in the reaction mixture was adjusted to 6-6.5 to prevent a removal of the cysteine protection group. In a next step, the remaining starPEG arms were conjugated to the basic leukocyte elastase responsive AAPV peptide by adding a 1.3 fold excess of peptide to the reaction mixture at pH 6-6.5. After complete reaction, the StBu protection group was cleaved by addition of TCEP in 6 fold excess. The conjugate was purified by preparative RP-HPLC. For coupling of the

maleimide functionalized compstatin 4W9A the conjugate and a 1.2 fold excess of drug were dissolved in DMF and reacted for 2h, followed by final conjugate purification by RP-HPLC.

Coupling of PMX53 derivative

A starPEG-peptide conjugate with full starPEG arm saturation by the leukocyte elastase responsive AAPV peptide was synthesized and purified as described in the Methods section. For the conjugation of the PMX53 derivative to the endstanding NH_2 of the responsive peptide, first 15 mmol L^{-1} starPEG-peptide conjugate were reacted with 1.2 mmol L^{-1} Maleimidopropionic acid N-hydroxysuccinimide ester in PBS (pH 7.4) for 1 h. In a second step, the PMX53 derivative was bound by Michael-type addition via its cysteine moiety. For this purpose, a final concentration of 1.2 mmol L^{-1} PMX53 derivative was added and the reaction mixture further incubated for 1 h, targeting a final drug conjugation degree of 2% starPEG-peptide arms. Subsequently, the reaction mixture was directly used for hydrogel formation. As control for stable hydrogel formation, four-armed starPEG amine was drug conjugated after the same principle.

Coupling of CsA

A starPEG-peptide conjugate with 90% starPEG arm saturation by the leukocyte elastase responsive AAPV peptide was synthesized and purified as described in the Methods section. The remaining starPEG arms were conjugated to the cysteine moiety of the MM284-(DE)$_6$-GKGAAPV-II-GGC-CONH$_2$ conjugate by Michael-type addition. For this purpose, the 90% saturated starPEG-peptide conjugate was reacted with 0.4 equal of drug for 1 h in PBS at pH 7.5-8, reaching a CsA conjugation degree of 10% starPEG-peptide arms. Subsequently,

starPEG-peptide conjugate with full starPEG arm saturation by the leukocyte elastase responsive AAPV peptide was added to the reaction solution in 4-fold excess, to finally result in 2% CsA conjugation degree per starPEG-peptide arm. The reaction mixture was directly used for hydrogel formation.

S1.3. Supplement – Basic analysis

Enzymatic cleavage of elastase-responsive peptide and PEG-AAPV conjugate

To analyze leukocyte elastase mediated hydrolysis of the responsive peptide and the corresponding conjugate, 7 mmol L^{-1} AAPV peptide or 0.7 mmol L^{-1} starPEG-AAPV conjugate were incubated with 20 nmol L^{-1} human neutrophil elastase in PBS, pH 7.4 at 37°C. The enzymatic reaction was evaluated after indicated timepoints by analytical HPLC, followed by integral analysis of the peak signals for educt and cleavage products.

Inhibition of leukocyte elastase by heparin

To evaluate the influence of the hydrogel component heparin on the activity of leukocyte elastase, samples with heparin sodium salt concentrations of 0.001-1 000 µg mL^{-1} were prepared in 50 mmol L^{-1} Tris-HCl pH 7.4 supplemented with 100 mmol L^{-1} NaCl and 0.1% BSA and pre-incubated with 10 nmol L^{-1} leukocyte elastase for 5 min. Subsequently, the cleavage of 250 µmol L^{-1} chromogenic substrate MeOSuc-Ala-Ala-Pro-Val-pNA by leukocyte elastase in the presence of the different heparin concentrations was measured as reaction kinetic at 37°C for 30 min using the Spark™ 10M multimode microplate reader (Tecan) at 405 nm and compared to a control without heparin.

Enzymatic cleavage of the fluorescent model drug system

Hydrogels with the fluorescent model drug (ɣ = 1.0) were prepared as described on 13 mm diameter PEMA coated glass slides, polymerized, and swollen in PBS for 24 h. After equilibration in 50 mmol L^{-1} Tris-HCl pH 7.4 supplemented with 100 mmol L^{-1} NaCl and 0.1% BSA for 30 min, gels were exposed to different concentrations of leukocyte elastase in 300 µLTris-BSA buffer at 37°C under constant shaking. As a control, mechanically comparable stable gels (ɣ=1.5) were incubated with the highest enzyme concentration. To follow hydrogel cleavage, 20 µL of the solution were removed at indicated timepoints and measured at $\lambda_{ex.}$=485 nm/ $\lambda_{em.}$= 535 nm against an Atto488 standard in Tris-BSA.
